# Leveraging Non‐Radiative Transitions in Asphaltenes‐Derived Carbon Dots for Cancer Photothermal Therapy

**DOI:** 10.1002/smll.202404591

**Published:** 2024-08-29

**Authors:** Ozioma Udochukwu Akakuru, Jie Xing, Shuqi Huang, Zubair M. Iqbal, Steven Bryant, Aiguo Wu, Milana Trifkovic

**Affiliations:** ^1^ Department of Chemical and Petroleum Engineering Schulich School of Engineering University of Calgary Alberta T2N 1N4 Canada; ^2^ Ningbo Key Laboratory of Biomedical Imaging Probe Materials and Technology Zhejiang International Cooperation Base of Biomedical Materials Technology and Application Chinese Academy of Sciences (CAS) Key Laboratory of Magnetic Materials and Devices Ningbo Cixi Institute of Biomedical Engineering Zhejiang Engineering Research Center for Biomedical Materials Ningbo Institute of Materials Technology and Engineering Chinese Academy of Sciences Ningbo 315201 China; ^3^ Institute of Smart Biomedical Materials School of Materials Science and Engineering Zhejiang Sci‐Tech University Hangzhou 310018 China

**Keywords:** asphaltenes, carbon dots, near infrared irradiation, non‐radiative transitions, photothermal therapy

## Abstract

Cancer photothermal therapy leverages the capability of photothermal agents to convert light to heat for cancer cell ablation and necrosis. However, most conventional photothermal agents (Au, CuS, Pd, mesoporous silica nanoparticles, and indocyanine green dye) either face scalability challenges or photobleached upon prolonged irradiation which jeopardizes practical applications. Here, asphaltenes‐derived carbon dots (ACDs, 5 nm) are rationally engineered as a low‐cost and photostable photothermal agent with negligible in vivo cytotoxicity. The abundant water‐solvating functional groups on the ACDs surface endows them with excellent water re‐dispersibility that outperforms those of most commercial nanomaterials. Photothermal therapeutic property of the ACDs is mechanistically described by non‐radiative transitions of excited electrons at 808 nm via internal conversions and vibrational relaxations. Consequently, the ACDs offer cancer photothermal therapy in mice within 15 days post‐exposure to one‐time near infrared irradiation. This pioneering study showcases the first utilization of asphaltenes‐based materials for cancer therapy and is expected to arouse further utilization of such materials in various cancer theranostics.

## Graphical abstract

Effective solvent pretreatment of asphaltenes precursor, succeeded by acid‐based oxidation pinpointed in engineering low‐cost, biocompatible, water re‐dispersible, and scalable asphaltenes‐derived carbon dots, which convert near infrared light to heat via non‐radiative transitions to provide cancer photothermal therapy.

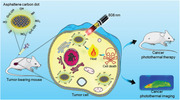

## 1 Introduction

Cancer is one of the diseases ravaging our society today and ranked top in the leading causes of death worldwide.^[^ ^1^ ^]^ Early diagnosis and treatment have been pinpointed as crucial strategies to reduce mortality rates.^[^ ^2^ ^]^ Although various established cancer treatment modalities such as chemotherapy, surgery, and immunotherapy exist,^[^ ^3^ ^]^ new treatment modalities continue to evolve. One example is near infrared (NIR)‐mediated photothermal therapy (PTT), which takes advantage of the transparency of biological tissues in the NIR region, enabling photothermal agents to effectively convert optical energy to heat energy with minimal invasiveness.^[^ ^4^ ^]^ NIR cancer photothermal therapy requires the use of conventional photothermal agents such as gold nanoparticles,^[^ ^5^ ^]^ CuS nanoparticles,^[^ ^6^ ^]^ conductive polymer nanoparticles,^[^ ^7^ ^]^ indocyanine green dye,^[^ ^8^ ^]^ that absorb light and convert same to heat to ablate cancer cells locally. Although the clinical translation of some of these photothermal agents has commenced in recent years, research is still ongoing to ameliorate the toxicity and photostability from the well‐known “melting effect” peculiar with metals.^[^ ^9^ ^]^ Polymeric nanoparticles and organic dyes on the other hand exhibit low photoconversion efficiency and photobleaching, respectively.^[^ ^10^ ^]^

Carbon dots, carbon‐based fluorescent nanoparticles with a size below 15 nm, have been recently discovered to possess photoconversion properties for cancer photothermal therapy.^[^ ^11^ ^]^ The low cytotoxicity and water dispersibility of hydrophilic carbon dots are huge drivers for their in vivo applications, especially in cancer treatment. For instance, hydrophilic carbon dots engineered from citric acid and urea were utilized to achieve cancer image‐guided photothermal therapy within 14 days post‐treatment.^[^ ^12^ ^]^ The carbon dots demonstrated good photostability up to five laser on/off cycles and expectedly caused no noticeable damage to major organs of mice, which shows their appreciable biocompatibility and potential for further biomedical applications. In another report, hydrophilic carbon dots were synthesized from watermelon juice via hydrothermal method and used for cancer photothermal treatment.^[^ ^13^ ^]^ The carbon dots exhibited negligible cytotoxicity, photostability up to 3 lasers on/off cycles, and induced substantial cancer cell death by one‐time laser irradiation. However, both carbon dots from watermelon juice^[^ ^13^ ^]^ and citric acid/urea^[^ ^12^ ^]^ precursors exhibit moderate hydrophilicity, and are engineered via hydrothermal and solvothermal methods at high temperatures (≥160 °C), with attendant low mass yield which jointly pose challenges to large‐scale production.^[^ ^12^, ^13^ ^]^ Engineering carbon dots of comparable cancer photothermal therapeutic properties and improved hydrophilicity, but with readily available precursors (e.g., asphaltenes) at low temperatures and appreciably high mass yield is desired.

Fossil and petroleum refining products such as coal, lignite, anthracite, coal tar pitch, and petroleum coke, have also been used to engineer carbon dots for metal‐ion sensing,^[^ ^14^ ^]^ bioimaging,^[^ ^15^ ^]^ photocatalytic hydrogen generation,^[^ ^16^ ^]^ and light emitting devices.^[^ ^17^ ^]^ Although asphaltenes present a low‐cost carbon precursor for engineering high‐value materials, most studies have been on carbon fibers derived from asphaltenes,^[^ ^18^ ^]^ with few reports on asphaltenes‐derived carbon dots (ACDs).^[^ ^19^ ^]^ Studies on the applications of ACDs are scarce and mainly report material synthesis and characterization.^[^ ^19^, ^20^ ^]^ Moreover, none of the reported ACDs have exhibited excellent water dispersibility compared to those recently developed in our laboratories,^[^ ^21^ ^]^ which is a highly sought after parameter for biomedical materials especially for cancer theranostics. Until now, there has been no report on the i) photothermal properties of any asphaltenes‐based material, and ii) utilization of any asphaltenes‐based material for cancer therapy.

Here, we report a low‐cost and scalable cancer photothermal agent based on ACDs benefiting from the cheap price and abundance of their asphaltenes precursor. The material synthesis protocol employed in this study ensures that the ACDs are benign as they do not induce noticeable cytotoxicity in vitro and to mice major organs after intravenous injection. The ACDs also show long‐term photostability and appreciable photoconversion properties which gives rise to cancer photothermal therapy by one‐time near infrared irradiation. Considering the intriguing ACDs properties of biocompatibility, photostability, and water re‐dispersibility, the ACDs are suitable low‐cost and scalable cancer photothermal therapeutic agents for clinical translation.

## 2 Results and Discussion

**Scheme 1:**
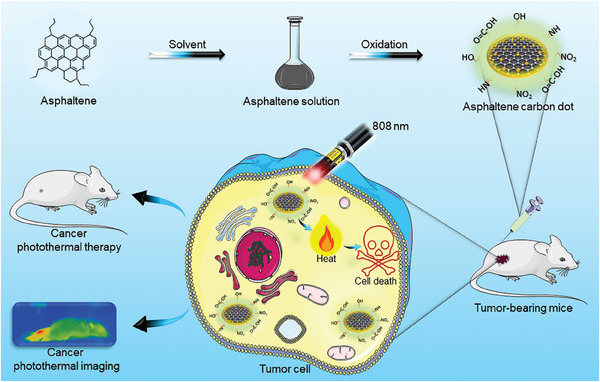
Schematic illustration of ACDs synthesis from asphaltenes precursor and subsequent application in cancer photothermal therapy.

A solvent pre‐treatment process entailing the use of toluene to dissolve asphaltenes was employed as a blueprint to improve the susceptibility of asphaltenes to oxidation with mixed acids including HNO_3_ and H_2_SO_4_ (**Scheme** **1**). This pre‐treatment process ensured the extensive oxidation of the asphaltenes to ACDs with abundant oxygen‐ and nitrogen‐based functionality responsible for the effective solvation of water molecules. Consequently, the ACDs are recovered at appreciably high mass yield (57%) and easily re‐disperse in water even after oven drying which is a property hitherto unattained in most commercial nanomaterials. The mass yield of ACDs can be compared to those of carbon dots synthesized from coal (20%),^[^ ^22^ ^]^ petroleum coke (55%),^[^ ^23^ ^]^ kitchen tea (9%),^[^ ^23^ ^]^ bee pollens (38%),^[^ ^24^ ^]^ blue‐green algea (57%),^[^ ^23^ ^]^ fruit bunch biochar (30%),^[^ ^25^ ^]^ coffee grounds (12%),^[^ ^26^ ^]^ egg (6%),^[^ ^27^ ^]^ banana juice (58%),^[^ ^28^ ^]^ graphite flakes (10%),^[^ ^29^ ^]^ and Chinese ink (73%).^[^ ^30^ ^]^

**Figure 1:**
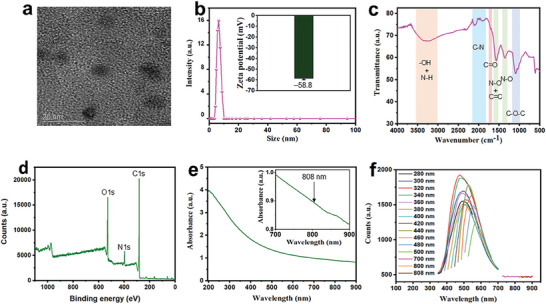
a) TEM image of the ACDs. b) Size and zeta potential of the ACDs dispersion in water. (c) FT‐IR spectra, d) XPS full survey, and e) UV–vis spectrum of the ACDs. The inset represents the absorbance of the ACDs in the NIR region. f) Fluorescence spectra of 0.02 mg mL^−1^ ACDs at various excitation wavelengths.

The transmission electron microscopy (TEM) image reveals the morphology of the ACDs and insight into the size of the particles (**Figure** **1**a). The particles show considerable dispersion without forming aggregates in water and could be generally described as nanodiscs. The dynamic light scattering (DLS) analysis shows hydrodynamic size of 5 nm in line with the particle size observation from the TEM images and corroborating the considerable water dispersibility of the ACDs (Figure 1b). The surface charge of the ACDs is ‒58.8 mV which could be attributed to the presence of high oxygen‐ and nitrogen‐based functional groups such as hydroxyl, epoxy, carbonyl, nitro, and amino groups. These functional groups are introduced into the ACDs by the extensive oxidation process with the mixed acids (HNO_3_/H_2_SO_4_). Consequently, the ACDs are endowed with impressive hydrophilicity and uniform fluorescence (absence of aggregation) in the 3D confocal image obtained with 275 mg mL^−1^ ACDs in water (Figure S1, Supporting Information). The ACDs are also stable in aqueous solutions using NaCl, CaCl_2_, and NaOH as model solutions, as colloidal stability is maintained up to 2 weeks of standing (Figure S2a, Supporting Information). This is confirmed by the absence of ACDs aggregation at high solute concentrations of 5% (Figure S2b, Supporting Information) and without significant change in their absorbance spectra (Figure S2c, Supporting Information).

Fourier transform infrared (FT‐IR) spectroscopy reveals the presence of the oxygen and nitrogen‐containing functional groups responsible for the excellent water re‐dispersibility of the ACDs. These groups include hydroxyl/amino, nitro, carbonyl, and ether groups with stretching vibrations at 3380, 1580–1356, 1711, and 1097 cm^−1^, respectively (Figure 1c). The X‐ray photoelectron spectroscopy (XPS) full survey further explains the abundance of the oxygen and nitrogen‐containing functional groups in the ACDs (Figure 1d). The considerable contents of oxygen (40.2%) and nitrogen (7.9%) show that most of the carbon atoms in the asphaltenes precursor are introduced with or converted to oxygen and nitrogen‐containing functional groups. The oxygen scan (O1s) portrays the different contents of oxygen‐based functional groups in the ACDs with the dominance of hydroxyl groups at 65% (Figure S3, Supporting Information). The nitrogen (N1s) and carbon (C1s) scans of the ACDs are shown in Figures S4 and S5 (Supporting Information), respectively.

Figure 1e shows the ACDs possess a broad absorption range (200–900 nm) between the ultraviolet‐visible (UV–vis) and NIR region. The absorbance of the ACDs in the NIR region (inset in Figure 1e) is similar to those of various graphene‐based nanomaterials,^[^ ^31^ ^]^ and carbon dots^[^ ^31^, ^32^ ^]^ that have been used for cancer photothermal therapy at 808 nm laser irradiation. The ACDs exhibit both *π*─*π* ^*^ and *n*─*π* ^*^ transitions corresponding to their sp^2^ and C–O conjugate carbon domains (*e.g*., carboxyl, hydroxyl), respectively. The *n*─π* and *π*─*π* ^*^ transitions agree with earlier observations in other ACDs derived by acid‐based oxidation in earlier reports.^[^ ^19^ ^]^ The ACDs also emit greenish blue luminescence upon exposure to a 365 nm UV lamp operated in the dark (Figure S6, Supporting Information). Fluorescence spectroscopy conducted on various concentrations of the ACDs in water shows a maximum emission wavelength of 472 nm (Figure 1f). It is observed that the light emission by the ACDs is dependent on the excitation wavelength, showing an initial blue shift from 200–320 nm, followed by a redshift as the excitation wavelength increases up to 500 nm. Importantly, low emission is observed between excitation wavelengths of 700 and 808 nm, suggesting that most of the absorbed light by the ACDs in those excitation wavelengths is converted to heat via non‐radiative transitions. This phenomenon is very crucial for the ACDs‐mediated cancer photothermal therapy upon 808 nm laser irradiation (*vide infra*). Information on other properties of the ACDs is contained in our recent study.^[^ ^21^ ^]^

**Figure 2:**
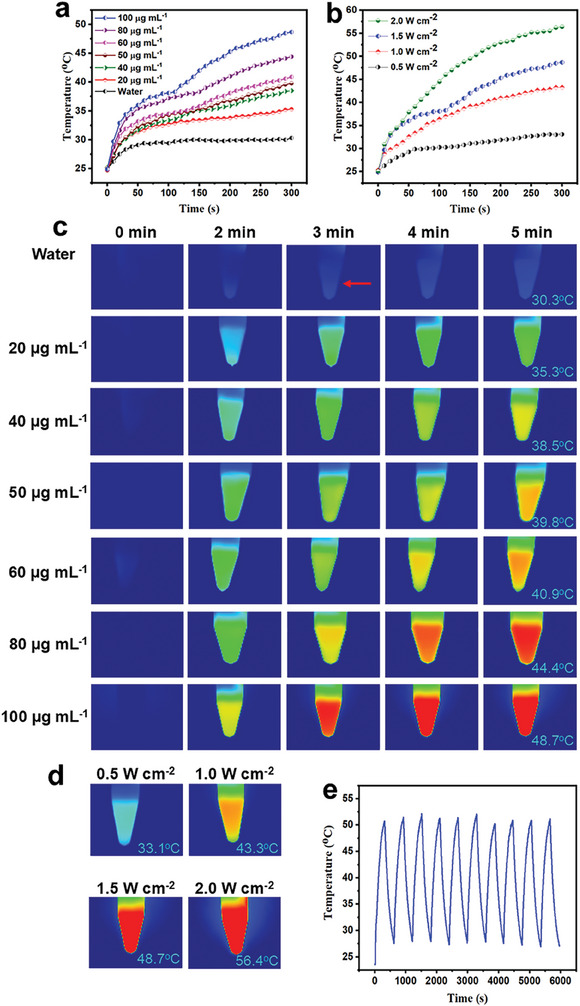
Temperature variations of ACDs at a) various concentrations using a laser optical power of 1.5 W cm^−2^ and b) various laser optical power densities using a fixed ACDs concentration of 100 µg mL^−1^. Laser wavelength is 808 nm and exposure time is 5 min. c) NIR photothermal images were generated with different concentrations of the ACDs with 1.5 W cm^−2^ laser power densities in 5 min. The red arrow shows the direction of laser irradiation. d) Photothermal images of 100 µg mL^−1^ ACDs exposed to 808 nm laser at various power densities (0.5, 1.0, 1.5, and 2.0 W cm^−2^). e) Temperature elevations of ACDs dispersion (100 µg mL^−1^) over 10 laser on/off cycles at 1.5 W cm^−2^.

To evaluate the capability of ACDs to induce cancer cell death by PTT, the in vitro photothermal behavior of different ACDs concentrations (20, 40, 60, 80, and 100 µg mL^−1^) were assessed on a digital NIR photothermal imaging system. The ACDs exhibit a concentration‐controlled temperature elevation profile upon exposure to an 808 nm NIR laser irradiation for 5 min (**Figure** **2**a). Temperature elevation with 1 mL of 100 µg mL^−1^ ACDs dispersion reaches 48.7 °C in 5 min. Contrarily, pure water serving as control shows marginal temperature elevation upon laser exposure (30.3 °C, 5 min). When the NIR laser optical power density is increased to 2.0 W cm^−2^, the temperature elevation could reach 56.4 °C in 5 min using 1 mL of 100 µg mL^−1^ ACDs dispersion (Figure 2b).

The preliminary photothermal imaging capability of the ACDs is established from the heat maps generated with different concentrations of ACDs dispersions in water after 5 min exposure to the 808 nm laser operated at 1.5 W cm^−2^ (Figure 2c). Photothermal images of 100 µg mL^−1^ ACDs exposed to the 808 nm laser at various power densities are shown in Figure 2d. Furthermore, the photostability of the ACDs was determined via laser on/off cycles. It is interesting to note that the ACDs exhibit near‐steady photostability for 10 laser on/off cycles using 1.5 and 1.0 W cm^−2^ laser optical power density with strong potential to maintain such impressive photostability above 10 laser on/off cycles (Figures 2e) and S7 (Supporting Information), respectively. This suggests that the ACDs could be used to generate prolonged heating of cancer cells in vivo to enhance cancer treatment efficacy. Such impressive photostability is a beneficial property of carbon dots that have been deployed for cancer photothermal therapy.^[^ ^12^, ^13^ ^]^ The photostability of photothermal agents based on polypyrrole nanoparticles is comparable to those of carbon dots.^[^ ^33^ ^]^ On the contrary, pristine gold nanorods which is one of the most investigated photothermal agents for cancer photothermal therapy show marked decline in photostability prior to the five laser on/off cycles.^[^ ^34^ ^]^ In another report on gold nanorods, up to a 20% decline in photostability was recorded in just four laser on/off cycles.^[^ ^35^ ^]^ The photostability of the ACDs also outperform those of the popular and commercially available indocyanine green dyes, whose photoconversion capability rapidly flattens off upon repeated laser on/off cycles.^[^ ^36^ ^]^

Photothermal conversion efficiency of ACDs at 808 nm obtained from Figure S8 (Supporting Information) is calculated to be 41.86%, which could be compared to those of polypyrrole,^[^ ^4a^ ^]^ iodinated polypyrrole,^[^ ^33b^ ^]^ indocyanine green‐polydopamine nanodiamonds,^[^ ^37^ ^]^ polyethylene glycol‐coated cyanine dyes,^[^ ^38^ ^]^ Au@Fe_3_O_4_@polypyrrole nanoparticles,^[^ ^39^ ^]^ Au nanoshells and Au nanorods.^[^ ^40^ ^]^

**Figure 3:**
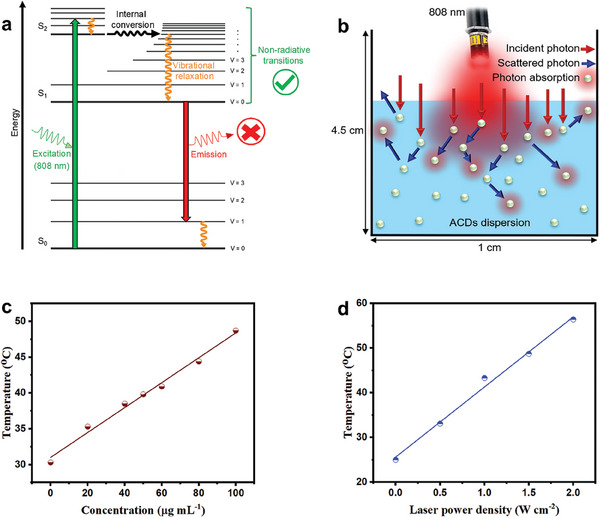
Schematic illustrations of a) non‐radiative transitions and b) possible light scattering by the ACDs upon 808 nm laser irradiation. c) Heat generation by different concentrations of the ACDs at 5 min upon 808 nm laser irradiation (1.5 W cm^−2^). d) Heat generation at 5 min using 100 µg mL^−1^ ACDs exposed to different power densities of the 808 nm laser.

The mechanism for heat generation for cancer PTT with carbon‐based nanoparticles has been largely described by the stimulation of loosely bound π electrons and subsequent relaxation of excited electrons.^[^ ^31^, ^41^ ^]^ In graphene quantum dots for instance, non‐radiative transitions is the major contributor to their low quantum yield.^[^ ^42^ ^]^ This is also the case for ACDs as they possess a low quantum yield of 2.04%. For a nanomaterial to exhibit low quantum yield, it implies that the majority of the energy absorbed by that nanomaterial at a particular wavelength is dissipated as heat rather than light emission. Considering that the ACDs show low light emission upon excitation at 808 nm (see Figure 1f), it is plausible that almost all energy absorbed by the ACDs at that wavelength is dissipated as heat via non‐radiative transitions (**Figure** **3**a). The generated heat in the process consequently increases the local temperature of the immediate surrounding of the ACD particles which can be leveraged to induce cancer PTT. In some other nanomaterials, light scattering can enhance heat generation.^[^ ^43^ ^]^ This was also considered for the ACDs and a possible light scattering mechanistic illustration for the ACDs is shown in Figure 3b. However, the heat generated at 5 min by different concentrations of the ACDs upon 808 nm irradiation (Figure 3c) and those generated at different laser power densities using a specific ACDs concentration (Figure 3d) is linear. For light scattering to occur, an increase in ACDs concentration for instance should cause a deviation from linearity in terms of heat generation possibly due to the reduced interparticle distance. These show that the possibility of light scattering by the ACDs to generate heat is very low. Therefore, non‐radiative electron relaxation including internal conversion and vibrational relaxation is pinpointed as the most probable mechanism for heat generation by the ACDs.

**Figure 4:**
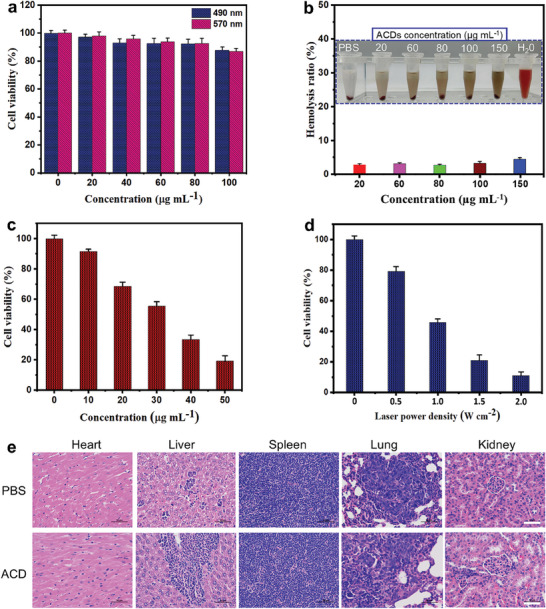
a) Cell viability of 4T1 cells co‐incubated with various concentrations of ACDs for 24 h. b) Hemolytic assay of the ACDs in red blood cells. Cell viability of 4T1 cells after 5 min treatment with c) different concentrations of ACDs under laser exposure at 1.5 W cm^−2^ and d) different laser optical power densities. Values are presented as mean ± standard deviation. e) H&E staining micrographs of mice major organs excised at 14 days post‐intravenous injection of PBS (control) and ACDs (200 µL, 2 mg mL^−1^). Scale bar = 50 µm.

Tissue tolerance is a critical parameter for materials intended for in vivo use. Moreover, considering the asphaltenes precursor of the ACDs, it is crucial to evaluate the biocompatibility of ACDs. The in vitro cytotoxicity of the ACDs was therefore evaluated in accordance with the conventional MTT (3‐(4,5‐dimethylthiazol‐2‐yl)‐2,5‐diphenyltetrazolium bromide) assay for biocompatibility assessment. After co‐incubation of mice breast cancer (4T1) cells with the ACDs for 24 h, cell viability of the cells is ≥87% even at 100 mg mL^−1^ ACDs (**Figure** **4**a). This agrees with the observation of biocompatibility in a previous study on ACDs.^[^ ^19b^ ^]^ Elemental analysis of the ACDs shows that the contents of noxious elements inherent in the asphaltenes precursor which could have jeopardized ACDs biocompatibility were substantially decreased after oxidation with the mixed acids. The biological safety of ACDs was further investigated by hemolytic assay. The ACDs show low toxicity (<5%) with red blood cells, suggesting minimum binding with red blood cells even at concentrations between 100 and 150 µg mL^−1^ (Figure 4b). It could be therefore inferred that the ACDs do not cause significant damage to cells and could be utilized as tissue‐tolerant nanoparticles for in vivo cancer therapy.

The in vitro cancer photothermal therapeutic performance of the ACDs was evaluated using 4T1 cancer cells alongside 808 nm laser irradiation by the MTT assay. Cancer cell death is caused by the ACDs after 808 nm laser irradiation (1.5 W cm^−2^) for 5 min with the effect intensifying as ACDs concentration increases (Figure 4c). Also, when a fixed concentration of the ACDs is used (50 µg mL^−1^), cancer cell mortality increases as the laser optical power density increases from 0.5 to 2.0 W cm^−2^ (Figure 4d). The in vitro therapeutic performance of the ACDs was visualized by live/dead cell staining assay. In the control, the cells are alive as seen from the green fluorescence. Substantial cell death is recorded with laser irradiation of the ACDs which could be visualized by the red fluorescence (Figure S9, Supporting Information). These findings indicate that the ACDs are effective at absorbing NIR light to generate sufficient heat levels capable of eradicating cancer cells.

To utilize the ACDs in vivo for cancer therapy, it is important to evaluate their in vivo cytotoxicity. Consequently, 200 µL of 2 mg mL^−1^ ACDs were injected into healthy mice and observed for 14 days. PBS served as the control. Hematoxylin and eosin (H&E) staining micrographs obtained from the main organs of mice (heart, kidney, lung, spleen, and liver) at 14 days post‐injection show no noticeable damage or inflammations to the organs compared with the control (phosphate buffered saline, PBS) group (Figure 4e). Nuclei chromatin and vacuole membranes distributions in the ACDs and PBS groups are comparable. Also, mice in both ACDs and PBS groups do not show abnormalities in their feeding, drinking, and movement habits.

Furthermore, the cellular uptake of the ACDs by 4T1 cancer cells was evaluated to determine whether the ACDs could be taken up by the cancer cells for therapeutic purposes. 4T1 cells were then co‐incubated with the ACDs for 4 h and the inherent fluorescence property of the ACDs was leveraged to acquire images of the ACDs in the cells. Figure S10 (Supporting Information) shows that the ACDs substantially accumulate in the cells. The results not only establish the 4T1 cellular uptake of the ACDs for cancer theranostics, but also suggest the possibility of using the ACDs to track cancer cells, leveraging their pronounced accumulation in the cell membranes.

**Figure 5:**
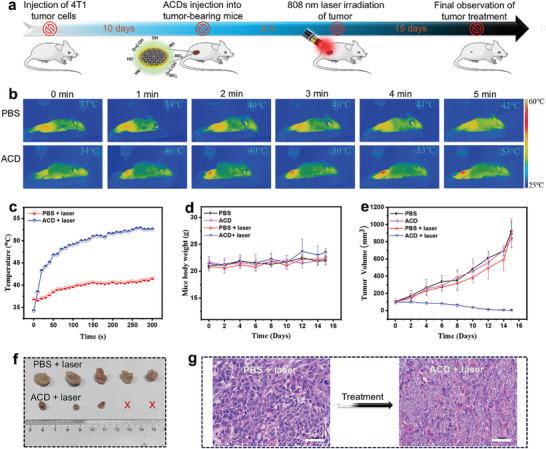
a) Schematic illustration of the timeline of mice PTT with the ACDs. b) Time‐course NIR photothermal images of whole mice revealing the tumor region upon laser irradiation (800 nm; 1.5 W cm^−2^) of the ACDs. PBS served as a control. c) Temperature elevations induced by the ACDs and PBS (control) in mice tumors during laser irradiation. d) Mice body weight and e) tumor volume changes monitored for 15 days post‐treatment with PBS and ACDs without and with laser exposure. Data are expressed as mean ± standard deviation. f) Representative digital images of the tumors excised from mice at 15 days post‐treatment with ACDs and PBS with laser exposure in each case (n = 5). g) H&E staining micrographs of tumors excised at 15 days post‐treatment with ACDs and PBS (control). Scale bar = 50 µm.

The promising in vivo biocompatibility, cellular uptake, in vitro cancer photothermal performance of the ACDs encourage their application for in vivo cancer PTT. Following inoculation of cancer cells in BALB/c mice, cancer PTT was evaluated according to the pattern shown in **Figure** **5**a. One‐time NIR irradiation was utilized to ensure protection of normal cells from hypothermia‐induced cell damage. The time‐course NIR photothermal images of whole BALB/c mice after injection with the ACDs enable detection of the cancer region by the heat maps generated from the ACDs in the cancer region (Figure 5b). Considering the small size (5 nm) and shape of the ACDs, there is possibility that they could penetrate the tumor cells by caveolae‐mediated endocytosis alongside penetration from tumor areas or normal tissue blood vessels. Since caveolae avoids the lysosomal degradation pathway, ACDs up taken via caveolae‐mediated endocytosis are less likely to be degraded, which preserves their functionality. This in turn ensures the accumulation of substantial amounts of ACDs in the cells to offer cancer PTT.

Figure 5c shows the local temperature elevations caused by the ACDs and PBS in the cancer region upon laser irradiation. The ACDs provide up to 53 °C rise in the local temperature of the tumor region which is sufficient to cause cancer cell death. Observation of the mice body weight over 15 days post‐treatment shows no significant change, suggesting that the mice behavior is relatively stable (Figure 5d). It also confirms that the ACDs do not induce noticeable cytotoxicity to mice. The tumor volume shows continuous decrease in the ACDs‐treated mice group contrary to the PBS only, ACDs only and PBS with laser groups (Figure 5e). After the 15‐day observation period, tumors in all the mice were excised to visualize the therapeutic efficacy. Digital image of the excised tumors shows substantial decrease in tumor volume in the ACDs with laser‐treated mice group contrary to the PBS with laser group (Figure 5f) and PBS only or ACDs only mice groups (Figure S11, Supporting Information). In two of the mice in ACDs group, the remaining tumors after treatment are too small to be excised, suggesting impressive photothermal therapeutic performance of the ACDs. Additionally, H&E staining micrographs of excised tumors at 15 days post‐treatment show pronounced tumor‐cell apoptosis in ACD‐treated mice compared with the PBS group (Figure 5g). The treatment time of 15 days achieved with the ACDs is similar with the time of 15 and 14 days required to treat tumor with gold nanoparticles,^[^ ^44^ ^]^ and carbon dots.^[^ ^12^ ^]^ Moreover, the various intrinsic properties of ACDs will enable their easy incorporation into other tumor treatment modalities such as immunotherapy, chemotherapy, and photodynamic therapy. Their excellent water dispersibility, biocompatibility, small size, facile synthesis, and variety of surface functionality are pinpointed to enable their incorporating with other therapeutic agents to improve cancer PTT efficacy and other treatment modalities.

## 3 Conclusion

We developed low‐cost and scalable ACDs that offer cancer PTT mediated by NIR irradiation. The low‐cost of the ACDs arises from their inexpensive and abundant asphaltenes precursor and the rational utilization of commercial grade reagents for the synthesis process. The utilization of mixed acids for ACDs synthesis endows them with abundant oxygen and nitrogen‐based functional groups to provide excellent water re‐dispersibility after oven drying. The ACDs also exhibit impressive tissue tolerance in vitro and in vivo and are effectively taken up but cancer cells to offer cell tracking. The hydrophilicity, biocompatibility, and cellular uptake of the ACDs qualify them for in vivo application in cancer theranostics. Upon exposure to one‐time NIR irradiation in mice, ACDs induce cancer cell death by PTT via heat generation from non‐radiative transitions. This study heralds the first application of asphaltenes‐based materials in cancer therapy and paves the way for the development of other asphaltene‐based systems for biomedical applications.

## Conflict of Interest

The authors declare no conflict of interest.

## Supporting information

Supporting Information

## Data Availability

The data that support the findings of this study are available from the corresponding author upon reasonable request.
