# Supplementary material for: Leveraging Non‐Radiative Transitions in Asphaltenes‐Derived Carbon Dots for Cancer Photothermal Therapy
Source: Small. 2024 Aug 29;21(10):2404591. doi: 10.1002/smll.202404591 (PMC11899496; doi:10.1002/smll.202404591)
Supplement: Supplementary file 1 — Supporting Information [file SMLL-21-2404591-s001.docx]

Copyright WILEY-VCH Verlag GmbH & Co. KGaA, 69469 Weinheim, Germany, 2016.

Supporting Information

*for Small*, DOI: 10.1002/smll. 202404591

Leveraging non-radiative transitions in asphaltenes-derived carbon dots for cancer photothermal therapy

Ozioma Udochukwu Akakuru^1^, Jie Xing^2^, Shuqi Huang^3^, Zubair M. Iqbal^3^, Steven Bryant^1^, Aiguo Wu^2^*, and Milana Trifkovic^1^*

^*^Emails: [mtrifkov@ucalgary.ca](mailto:mtrifkov@ucalgary.ca) (M. Trifkovic); aiguo@nimte.ac.cn (A. Wu)

**Experimental Section**

*Materials:* Powder asphaltenes were supplied by InnoTech Alberta, Canada. HNO_3_, H_2_SO_4_, and NaOH were purchased from Sigma Aldrich (Canada). De-ionized (DI) water (Millipore Milli-Q grade; resistivity = 18.2 MΩ) was used in all experiments. All chemicals were used as received without further purification.

*Synthesis of ACDs:* Solvent pre-treatment protocol was first employed, 1 g of asphaltenes powder was dissolved in toluene. Thereafter, 2.5 mL each of HNO_3_ and H_2_SO_4_ (70% and 98% aqueous solutions, respectively) was added to the residue. The mixture was stirred at 80^o^C for 4 h and was allowed to cool to room temperature. Thereafter, the mixture was centrifuged at 3000 rpm for 10 min and this process was repeated three times to remove the acid components. The recovered product was dispersed in water and brought to neutral pH with few drops of 0.1 M NaOH. The product was further purified by dialysis in a dialysis membrane (molecular weight cut off = 3500 Da) against DI water for 2 days with daily change of water. The super hydrophilic ACDs were then obtained as a fine black powder after vacuum drying for 48 h at 50^o^C and used for characterization and friction reduction without post-synthesis modification.

*Characterization:* ACDs morphology was investigated on a JEOL JEM-ARM200cF transmission electron microscope (TEM), which was operated at a 200 kV accelerating voltage. The microscope was equipped with a cold field-emission gun and a probe spherical aberration corrector. Dynamic light scattering (DLS) and zeta potential distribution of the ACDs were measured on a Zetasizer Nanoseries device (Nano-ZS, Malvern Instruments, Britain) to determine the sizes and surface charges of the nanoparticles, respectively. The functional groups in pristine asphaltenes and ACDs were determined by X-ray photoelectron spectroscopy (XPS) using Kratos Axis Ultra XPS spectrometer (Kratos Analytical, USA), and Fourier transform infrared (FT-IR) spectroscopy conducted on an Agilent Cary 630 FTIR Spectrometer (Agilent Instruments, USA). Elemental analysis of pristine asphaltenes and ACDs was conducted on an Agilent 8900 inductively coupled plasma mass spectrometer (Agilent Instruments, USA). Dispersibility of the ACDs was assessed by confocal microscopy on a Leica DMi8 (Leica Microsystems, USA) at 405 nm excitation wavelength.

*Photothermal effects:* Different concentrations (20, 40, 60, 80, and 100 µg mL^-1^) of ACDs dispersed in 1 mL of de-ionized water were irradiated with a laser (808 nm, 1.5 W/cm^2^) for 5 min in accordance with a previous method.^[1]^ To study the effect of optical density on photothermal conversions, 1 mL of 100 µg mL^-1^ ACDs dispersion was irradiated for 5 min with the 808 nm laser operated at various power densities (0.5, 1.0, 1.5, and 2.0 W cm^-2^). De-ionized water was used as control in the experiments. The photostability of 1 mL 100 µg mL^-1^ ACDs was determined in an 'on/off' cycle experiment by irradiation with the 808 nm laser (1.5 W cm^-2^) for 5 min (laser ON) and naturally cooling to room temperature (laser OFF). The photostability was tested by repeating the cycle ten times. In all cases, corresponding temperature changes were simultaneously recorded every 10 s using a digital thermometer with a thermocouple probe.

*Photothermal conversion efficiency:* The photothermal conversion efficiency (η) of the ACDs was determined according to the reported method. ^[2]^ In a typical experiment, ACDs solution (100 µg mL^-1^) was irradiated with an 808 nm laser (1.5 W cm^-2^) until the temperature of the solution reached a steady state. Then, the laser was turned off and the solution was allowed to naturally cool to the ambient temperature. The photothermal conversion efficiency (η) was then calculated according to equation (1).

$\eta= \frac{hs\left( T_{max} -T_{surr} \right) - Q_{0}}{I(1-{10}^{A_{\lambda}})}$ (1)

where *h* is the heat transfer coefficient and *s* represent the surface area of the container. The value of *hs* was gained from the plot of linear time data versus –Ln(θ) obtained from the cooling period of ACDs. *T*_max_ − *T*_surr_ is the temperature change of the ACDs solution at the maximum steady-ambient temperature. *Q*_0_ represents heat dissipated from light absorbed by the solvent and the container. *I* represent the power density of the laser and *A_λ_* is the absorbance of ACDs at 808 nm.

*Cell culture and in vitro cytotoxicity assay:* Mouse breast cancer (4T1) cell lines were cultured in RPMI-1640 medium supplemented with 10% FBS, 100 mg/mL streptomycin (Keygene) and 100 unit per mL penicillin, at 37°C in a 5% CO_2_ humidified atmosphere. The *in vitro* cytotoxicity of the ACDs was determined by MTT assay.^[3]^ Typically, 100 µL 4T1 cells were separately seeded into 96-well culture plates at 1 × 10^5^ cells per mL and cultured for 24 h at 37°C in 5% CO_2_. The cells were then washed thrice with phosphate-buffered saline (PBS, pH=7.4) and subsequently incubated alongside different concentrations (20, 40, 60, 80, and 100 µg mL^-1^) of ACDs for 24 h. Thereafter, 10 µL of 5 mg mL^-1^ MTT solution was added to each well and further incubated at 37°C in 5% CO_2_ for 4 h. Then, the growth medium was removed, followed by the addition of 100 μL DMSO to each well to dissolve the formazan crystals. The cells were co-incubated with the materials and MTT solution for another 30 min. Thereafter, absorbance of the resulting solutions in the wells was measured at 490 nm using a microplate reader (iMark 168-1130, Bio-rad, USA). The control group contained 4T1 cells and culture medium without the ACDs. Cell viability was then calculated as a percentage of the control samples according to equation (2).^[4]^

Cell viability (%) = [A]sample/[A]control × 100 (2)

where [A]sample and [A]control represent the absorbance of formazan products in the treated and untreated cells, respectively.

*In vitro cancer PTT:* To investigate the therapeutic ability of the ACDs, 100 μL of 4T1 cells (1 × 10^5^ per mL) were separately incubated in 96-well plates for 24 h in RPMI-1640 medium.^[5]^ Then, 100 μL of fresh media or 100 μL media containing various concentrations (10, 20, 30, 40, and 50 µg mL^-1^) of the ACDs were used to replace the old cell culture medium and incubated for another 24 h. After washing with PBS several times, 100 μL of fresh cell culture medium was added into the cells. Subsequently, the cells were irradiated for 5 min with an 808 nm laser operated at 1.5 W cm^-2^. To study the effect of optical density on therapeutic efficacies, the concentration of the ACDs was kept constant at 50 µg mL^-1^ in freshly prepared 96-well plates with 4T1 cells (1 × 10^5^ per mL), while laser power density was varied (0.5, 1.0, 1.5, and 2.0 W cm^-2^). Absorbance of the resulting solutions in each well was measured at 490 nm with the microplate reader.

To visually assess the photothermal therapeutic performance of the ACDs, live/dead cell co-staining experiments were conducted with Calcein-AM/ propidium iodide double stain kit (Yeasen) according to a previous method.^[1]^ In a typical experiment, 100 μL cells of 4T1 cells (1 × 10 5 cells per mL) were incubated in a 96-well plate for 24 h. Then the culture medium was replaced with 100 μL of RPMI-1640 medium containing ACDs and incubated for another 24 h. After washing with PBS for several times, 100 μL of RPMI-1640 medium was added to the cells. Thereafter, the cells were irradiated with 808 nm laser (1.5 W cm^-2^) for 5 min. The PBS group (control) was irradiated under the same condition. After laser irradiation, the cells were further cultured for 24 h and co-stained by Calcein-AM/propidium iodide working solution for 15 min at 37 °C. The viability of cells was evaluated by fluorescence microscopy.

*Cellular uptake:* For the cellular uptake tests, 1 × 10^5^ per mL of 4T1 cells were incubated in glass coverslips for confocal imaging with corresponding cell culture medium and cultured for 24 h at 37°C in a 5% CO_2_ humidified atmosphere.^[1]^ Thereafter, the cell culture medium was carefully removed and the cells were washed repeatedly with PBS solution (pH=7.4). Then, 1.0 mL of ACDs (1.0 mg/mL diluted in the cell culture medium) was added to cells in the glass coverslips and incubated. After 4 h, the cell culture medium with the ACDs were removed and cells were washed thrice with PBS, and fixed with 1 mL (4%) paraformaldehyde at 4^o^C for 5 min. After that, the cells were treated with 1 mL (0.5%) triton X-100 for 5 min, followed by 1 mL (1%) BSA for 30 min at room temperature. Finally, the cells were washed twice with PBS, and finally kept in 1 mL of PBS. Imaging of the cells by LCSM was conducted at excitation wavelength of 405 nm and emission wavelengths between 485‒600 nm. Fluorescence intensity of ACDs in the 4T1 cells was quantified by ImageJ software.

*Hemolysis test*: Venous blood was collected from the eyeball of healthy mice in anticoagulant tubes containing EDTA. The blood sample was shaken evenly and centrifuged at 3000 rpm for 15 min to obtain blood cells to evaluate ACDs hemolysis rate. Different concentrations of ACDs (20, 60, 80, 100, and 150 µg mL^-1^) was prepared and 1 mL of each ACDs solution, 1 mL of ultrapure water and 1 mL of PBS solution were mixed with 20 µL of blood cells. This was followed by incubation at 37°C for 4 h, then centrifugation at 3000 rpm for 15 min. A microplate reader then was used to measure the absorbance of the supernatant of each sample at 542 nm.

*Establishment of 4T1 tumor xenograft:* All animal experiments were carried out with the approval of the Regional Ethics Committee for Animal Experiments of Ningbo University, China [Permit No. (Zhe) 2019-0005]. Mice that were used for all in vivo experiments in this work were purchased from the Kawensi Biological Products Sales Center (Nanjing, China). 4T1 cells (1 × 10^5^, 100 μL) suspended in RPMI-1640 growth medium were subcutaneously injected into the back (right flank region) of the female Bagg Albino (BALB/c) mice (17-19 g, aged 5-6 weeks) according to a previous method.^[6]^

*In vivo biocompatibility assay:* To evaluate the biocompatibility of the ACDs *in vivo*, healthy nude mice were divided into two groups (n=5) in accordance with the method employed by Zeng et al.^[7]^ The first group was intravenously injected with 200 μL of 2 mg mL^-1^ ACDs, while the second group was intravenously injected with 200 μL PBS (pH=7.4) as control. Changes in body weight and behavior such as feeding and drinking habits were monitored carefully in the mice groups for the 15-day observation period. After 15 days, all mice were euthanized, and their major organs (heart, liver, lung, kidney, and spleen) were excised followed by fixing with 4% paraformaldehyde for the histological analysis by Hematoxylin and Eosin (H&E) staining. The slides were observed under an optical microscope.

*In vivo cancer photothermal imaging and PTT:* The imaging and therapeutic experiments were performed when the tumor volume reached about 130 mm^3^, in accordance with an already established procedure.^[1]^ Typically, 50 µL of PBS as control, and 50 µL of 2 mg mL^-1^ ACD was injected into 4T1 tumor-bearing mice. Photothermal imaging was conducted with near infrared laser irradiation of the tumor region. PTT of the mice was then observed for a period of 15 days post-irradiation. The mice were randomly divided into two groups (n=5): (1) PBS + 808 nm laser group, and (2) ACDs + 808 nm laser group. The day of laser exposure was set as day 0. Body weights and tumor volumes were recorded over the 15-day observation period. The tumor volumes (mm^3^) were calculated using the ellipsoid volume equation according to equation (3) ^[8]^.

V = (π/6) × (d^2^ × D) (3)

where d and D indicate the minor and major tumor axes, respectively.

*Statistical Analysis*: Data were expressed as mean ± standard deviation.


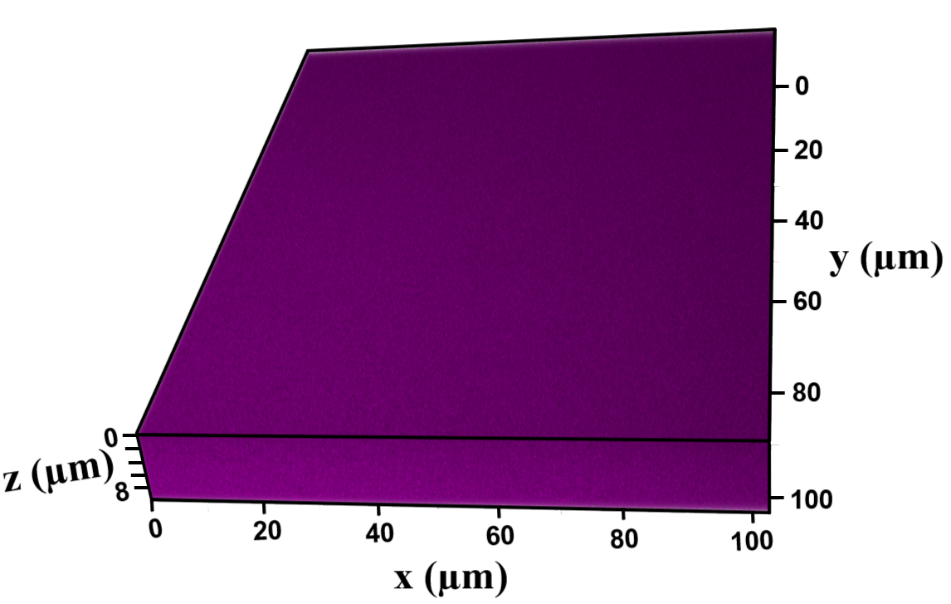


**Figure S1**. 3D confocal microscopic image of 275 mg mL^-1^ ACDs dispersion in water.


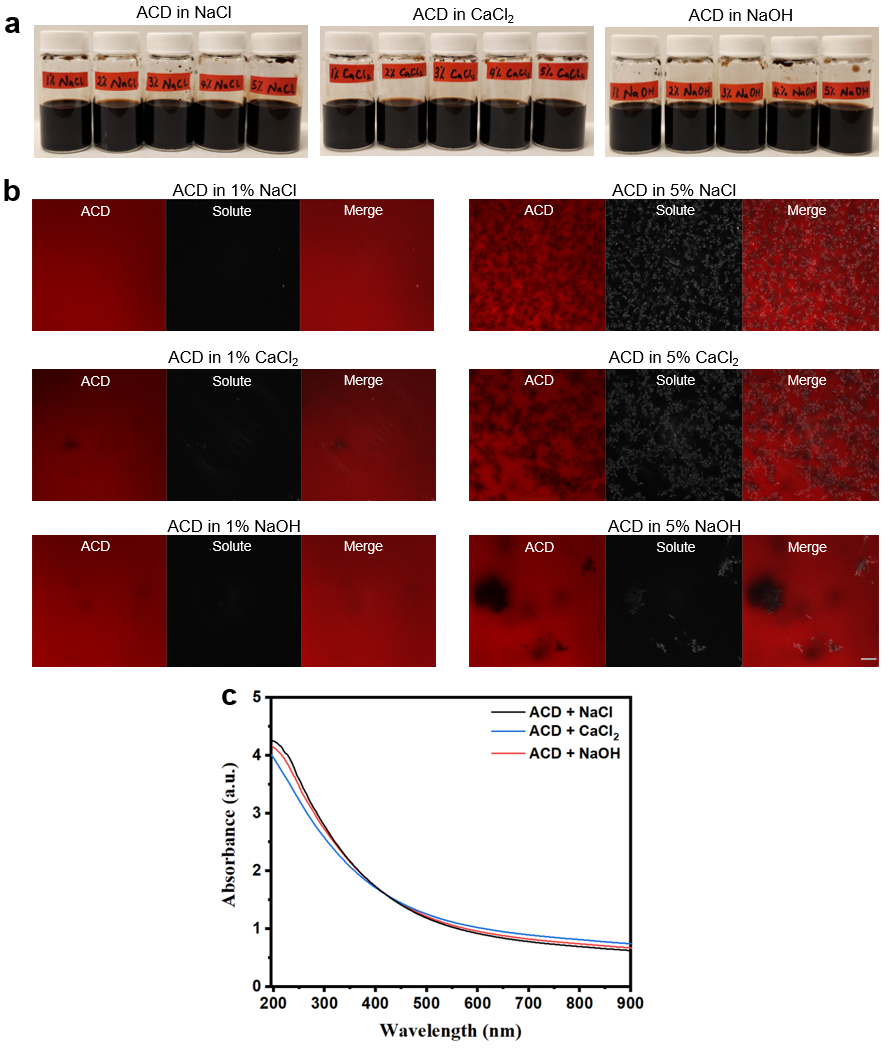


**Figure S2**. (a) Digital photo of vials containing ACDs dispersed in aqueous solutions of NaCl, CaCl_2_, and NaOH after 2 weeks showing lack of phase separation. (b) Confocal microscopy images of ACDs in 1% and 5% NaCl, CaCl_2_, and NaOH as representative solutions obtained after 2 weeks. Scale bar = 20 µm. (c) UV-vis spectra of ACDs in 5% aqueous solutions after 2 weeks.


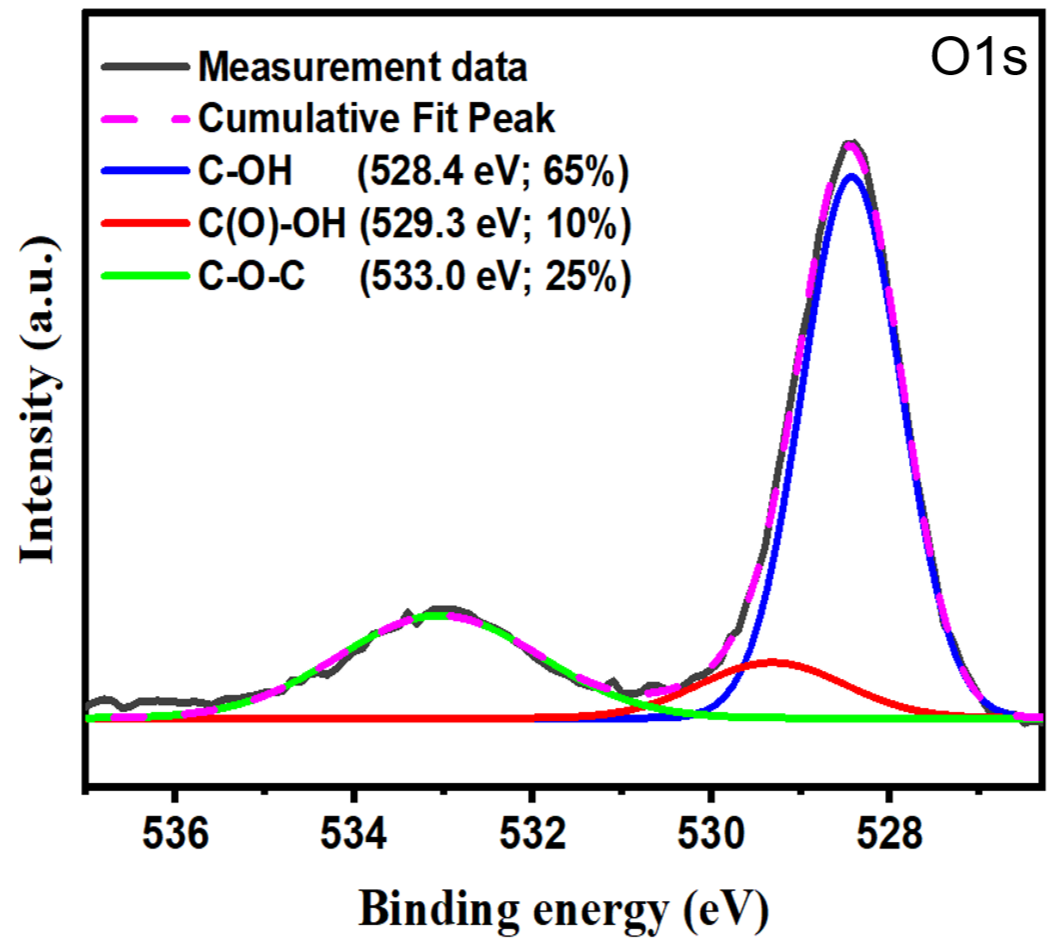


**Figure S3**. O1s XPS scans of the ACDs.


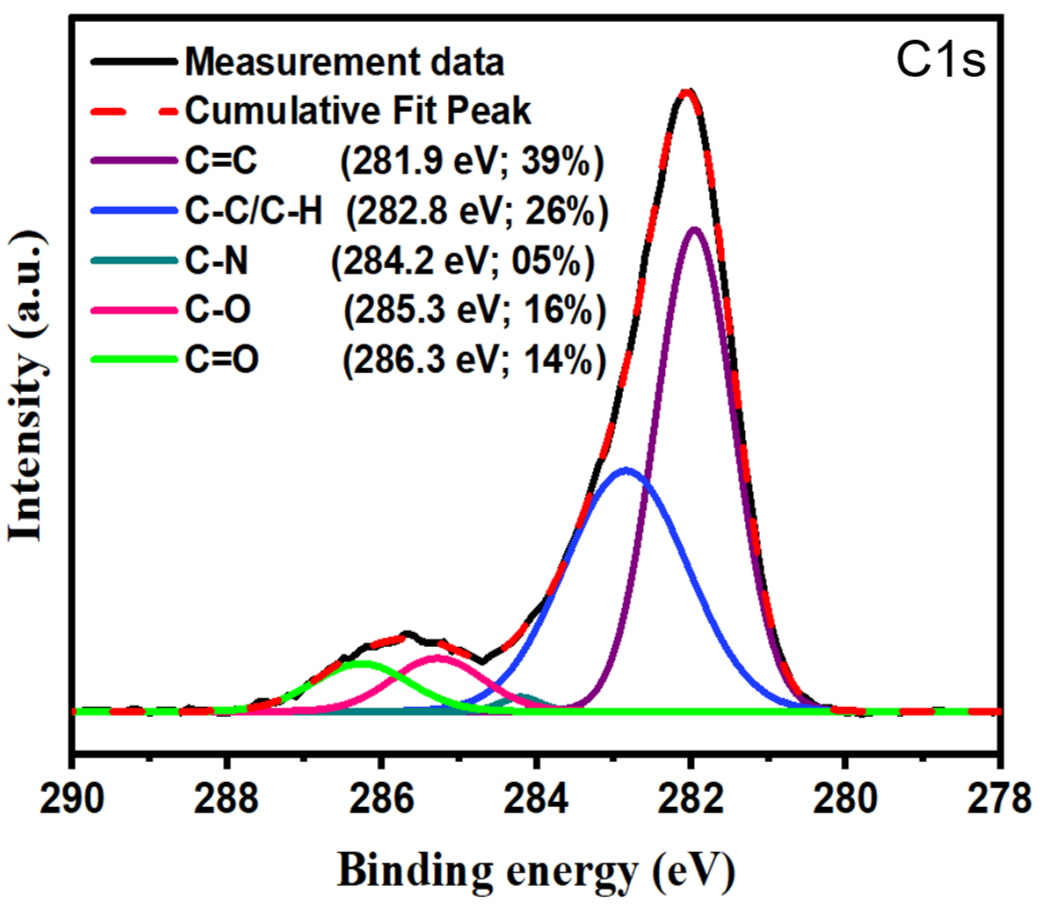


**Figure S4**. C1s XPS scans of the ACDs.


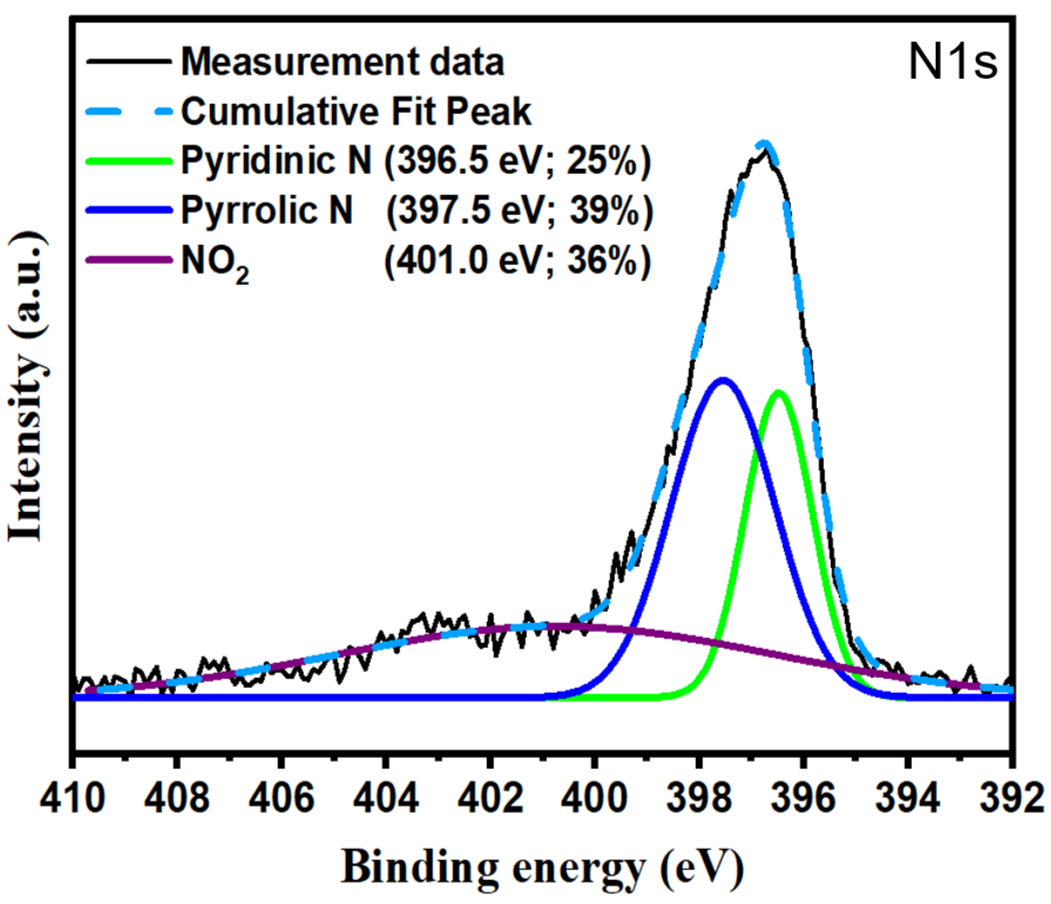


**Figure S5**. N1s XPS scans of the ACDs.


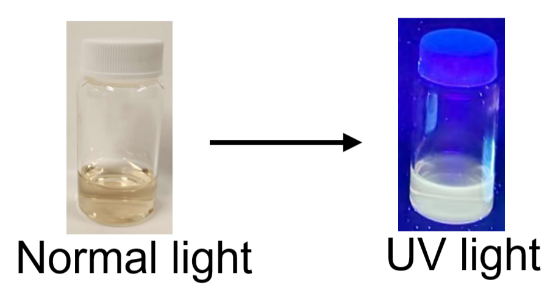


**Figure S6**. Photoluminescence of ACDs upon exposure to 365 nm UV lamp in the dark.


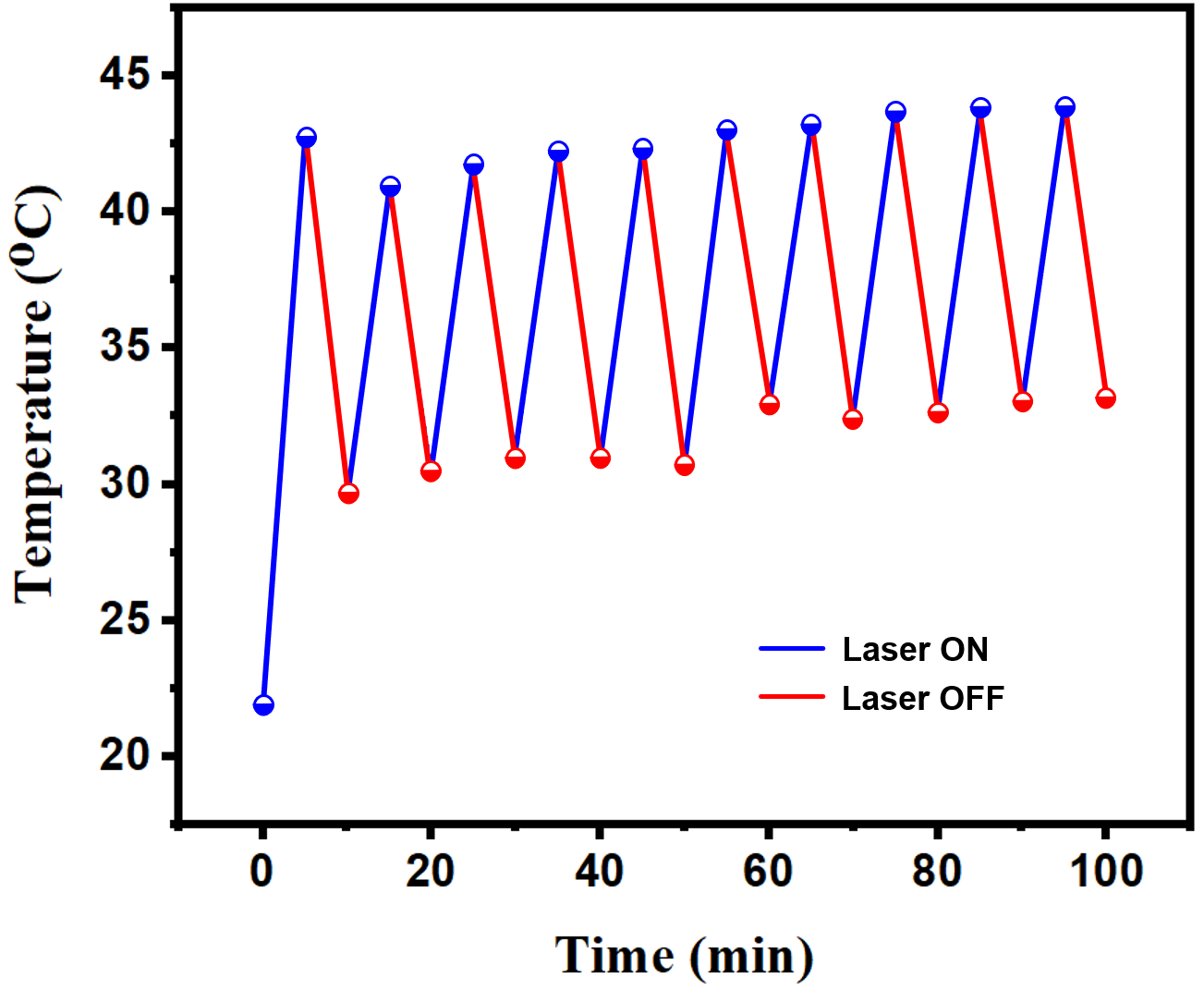


**Figure S7**. Temperature elevations of ACDs dispersion (100 µg mL^-1^) over 10 laser on/off cycles at 1.0 W cm^-2^.


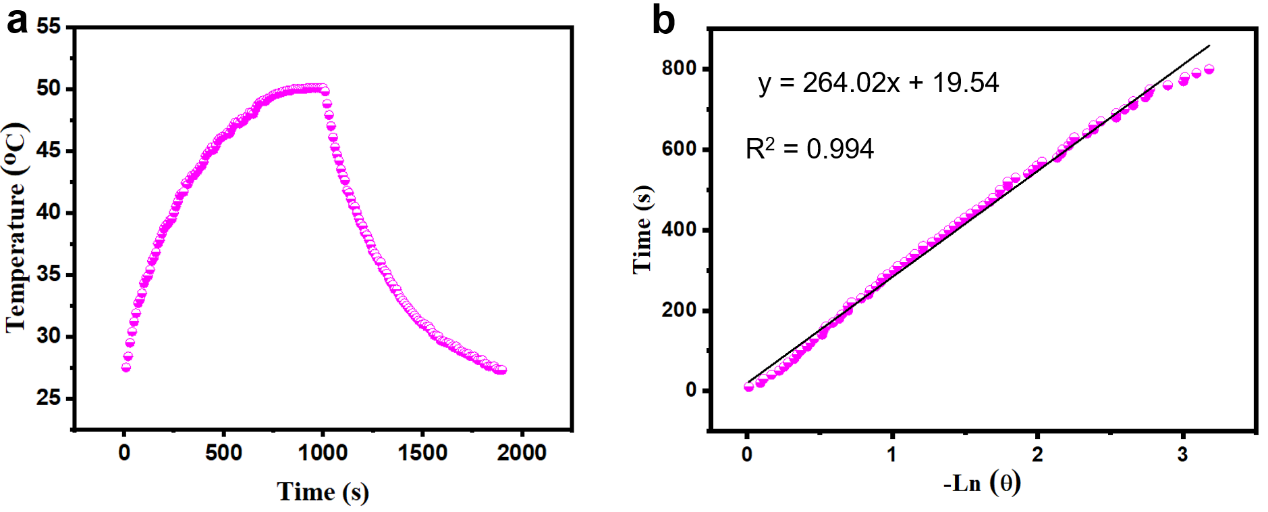


**Figure S8**. (a) Temperature change curve and (b) linear time data versus –ln(θ) obtained from the cooling period of ACDs.


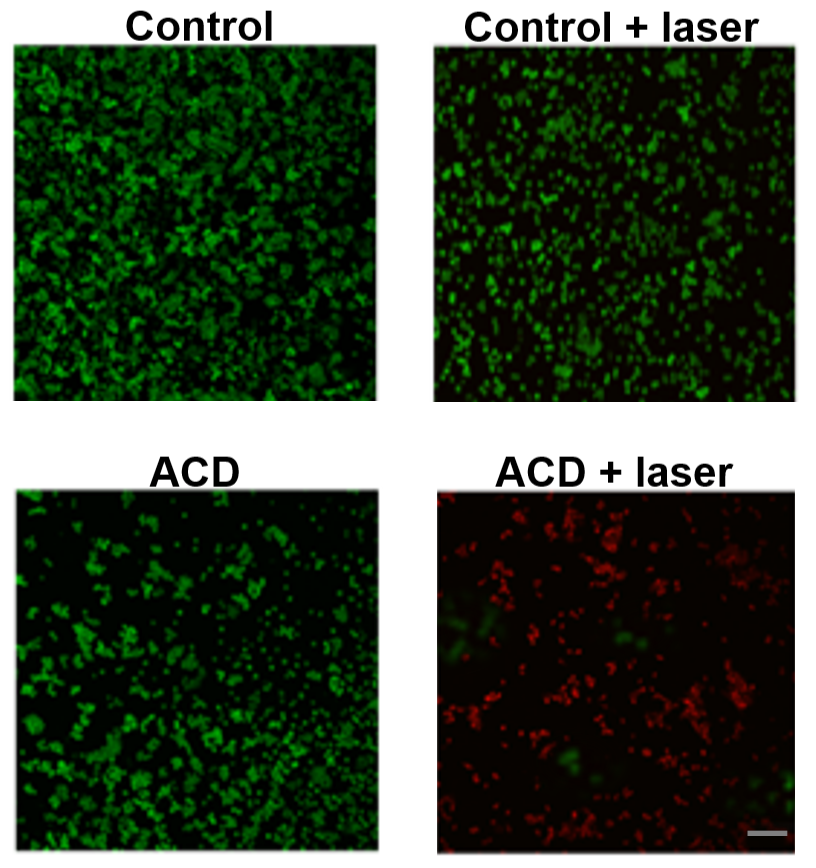


**Figure S9**. Microscopic images of ACDs incubated with 4T1 cells and irradiated with an 808 nm laser for 5 min. The cells were co-stained by calcein-AM and propidium iodide before imaging, scale bar: 100 μm.


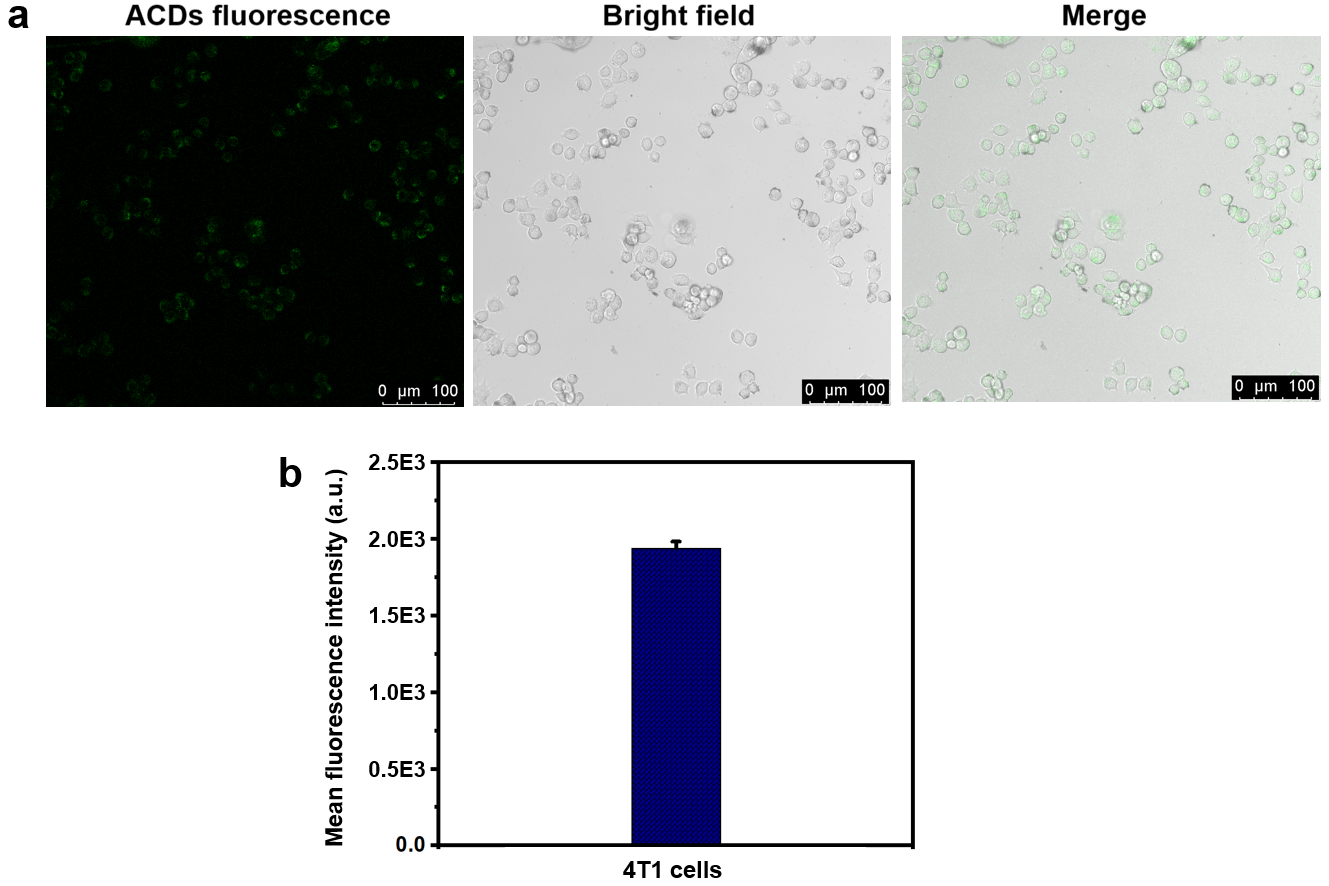


**Figure S10**. (a) Laser confocal fluorescence images of 4T1 cells co-incubated with ACDs for 4 h. (b) Quantitative analysis of the fluorescence signal intensity of ACDs in the cells.


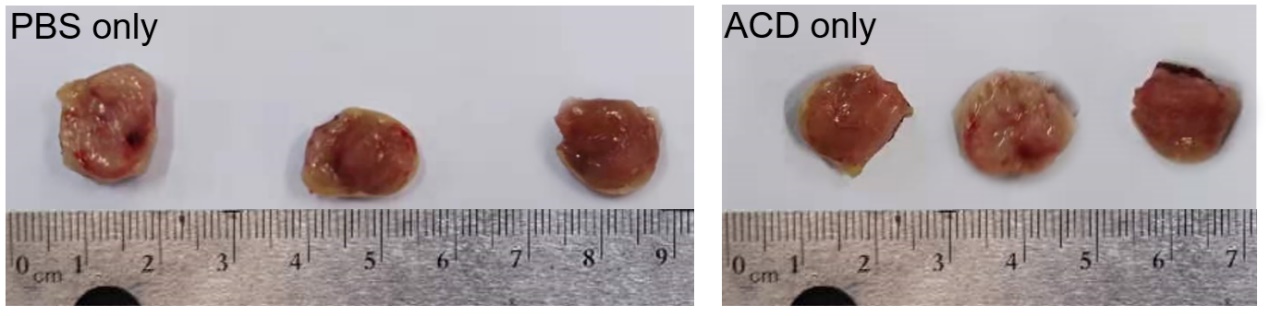


**Figure S11**. Representative digital images of the tumors excised from mice at 15 days post-treatment treated with PBS and ACDs without laser exposure.

**Supporting References**

[1] C. Liu, L. Luo, L. Zeng, J. Xing, Y. Xia, S. Sun, L. Zhang, Z. Yu, J. Yao, Z. Yu, O. U. Akakuru, M. Saeed, A. Wu, *Small* **2018**, *14*, 1801851.

[2] Y. Liu, K. Ai, J. Liu, M. Deng, Y. He, L. Lu, *Advanced Materials* **2013**, *25*, 1353-1359.

[3] G. Potsi, A. B. Bourlinos, V. Mouselimis, K. Poláková, N. Chalmpes, D. Gournis, S. Kalytchuk, O. Tomanec, P. Błoński, M. Medveď, P. Lazar, M. Otyepka, R. Zbořil, *Applied Materials Today* **2019**, *17*, 112-122.

[4] C. Chen, N. Kang, T. Xu, D. Wang, L. Ren, X. Guo, *Nanoscale* **2015**, *7*, 5249-5261.

[5] M. Z. Iqbal, W. Ren, M. Saeed, T. Chen, X. Ma, X. Yu, J. Zhang, L. Zhang, A. Li, A. Wu, *Nano Research* **2017**.

[6] O. U. Akakuru, M. Z. Iqbal, C. Liu, J. Xing, Z. Wei, Z. Jiang, Q. Fang, B. Yuan, E. I. Nosike, J. Xia, Y. Jin, J. Zheng, A. Wu, *Applied Materials Today* **2020**, *18*, 100524.

[7] L. Zeng, L. Luo, Y. Pan, S. Luo, G. Lu, A. Wu, *Nanoscale* **2015**, *7*, 8946-8954.

[8] C. Shirata, J. Kaneko, Y. Inagaki, T. Kokudo, M. Sato, S. Kiritani, N. Akamatsu, J. Arita, Y. Sakamoto, K. Hasegawa, N. Kokudo, *Scientific Reports* **2017**, *7*, 13958.
